# Bilateral adrenal hemorrhage after pancreaticoduodenectomy

**DOI:** 10.1093/jscr/rjad411

**Published:** 2023-07-31

**Authors:** Sarah Andres, Nikki Suchak, Maureen Brady, Moshim Kukar, Joy Sarkar

**Affiliations:** Jacobs School of Medicine and Biomedical Sciences, The State University of New York, Buffalo, NY 14203, USA; Nichols School, Buffalo, NY 14216, USA; Department of Surgical Oncology, Roswell Park Comprehensive Cancer Center, Buffalo, NY 14203, USA; Department of Surgical Oncology, Roswell Park Comprehensive Cancer Center, Buffalo, NY 14203, USA; Department of Surgical Oncology, Roswell Park Comprehensive Cancer Center, Buffalo, NY 14203, USA

**Keywords:** bilateral adrenal hemorrhage, pancreaticoduodenectomy, Whipple, adrenal insufficiency, postoperative

## Abstract

The incidence of bilateral adrenal hemorrhage (BAH) in the postoperative setting is rare, but potentially life threatening. A literature review of postoperative BAH reveals that there is limited data on BAH following abdominal surgery. We present a case of BAH following pancreaticoduodenectomy, which has not been previously documented in the literature. A 70-year-old male patient with no previous history of adrenal disease underwent an uncomplicated pancreaticoduodenectomy and was discharged after a typical postoperative course. He was readmitted with abdominal pain and ileus on POD 8 and a computed tomography (CT) scan was initially unremarkable, but a repeat CT scan on POD 11 demonstrated BAH. He was found to have adrenal insufficiency and was successfully treated with steroids. Clinicians should be aware of the possibility of adrenal hemorrhage postoperatively as it can potentially be a fatal surgical complication. To enhance patient outcomes, early detection and appropriate treatment are essential.

## INTRODUCTION

Bilateral adrenal hemorrhage (BAH) is an uncommon but serious condition when associated with adrenal insufficiency (AI). Reported precipitating conditions include neoplasm, coagulopathy and sepsis [1, 2]. Even in the presence of risk factors, BAH is rare, with incidence between 0.14 and 1.8% [1–3]. Abdominal surgery has been implicated in adrenal hemorrhage (AH) as well [1, 4–7] in ⁓10% of AH [8]. There are no reports of AH post-pancreaticoduodenectomy (Whipple procedure).

Though uncommon, BAH can lead to rapid decompensation [1]. Historically, a mortality rate of 27.8% has been reported in addition to a 100% mortality rate in unrecognized AI [3]. In severe hemorrhage, adrenalectomy may be required [2].

We present the first reported case of post-Whipple BAH, which manifested with abdominal pain and electrolyte abnormalities.

## CASE REPORT

A 70-year-old male nonsmoker with strong family history of pancreatic cancer and personal history of idiopathic pancreatitis was found to have a 17 mm pancreatic head mass with pancreatic ductal dilation on screening endosonography (EUS). Fine needle aspiration returned highly atypical cells; malignancy could not be excluded.

He had no prior abdominal operations. He denied alcohol use, jaundice or symptoms of pancreatitis.

CA 19-9 and carcinoembryonic antigen (CEA) were normal. Computed tomography (CT) scan findings correlated with EUS. Of note, both adrenal glands were radiographically normal.

He was offered open pancreatoduodenectomy for diagnosis and potential cure.

A prophylactic dose of heparin was administered on the morning of surgery. The operation was completed in a standard fashion without complication and drains were placed abutting the pancreatojejunostomy and hepaticojejunostomy. Estimated blood loss was 200 mL and total time was 374 min.

Pathology revealed intraductal mucinous papillary neoplasm with high grade dysplasia and negative margins. All 14 lymph nodes were normal (pTisN0).

His immediate postoperative course was routine. He had no evidence of biochemical pancreatic leak and drains were removed. He was discharged on POD 7 at which time he was afebrile, stable, tolerating diet, with pain well controlled.

**Figure 1:**
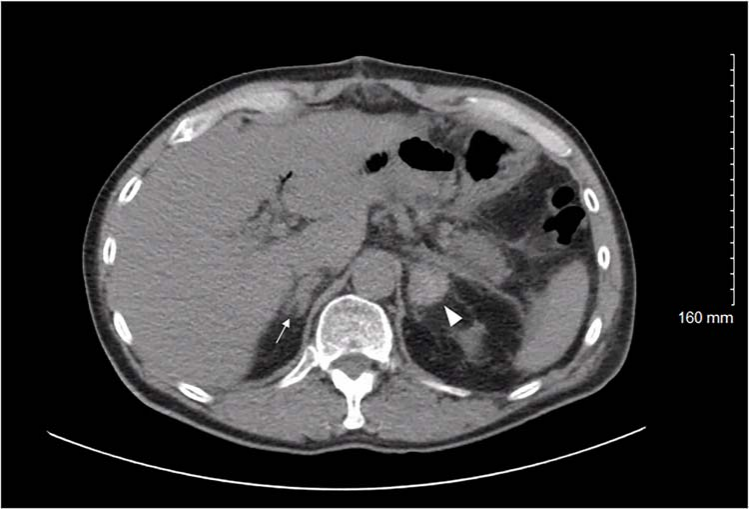
Axial CT abdomen/pelvis on POD 11 demonstrating the left adrenal gland (white arrowhead) with a high density 2.6 × 2.6 cm fluid collection; density is consistent with a subacute bleed. The right adrenal gland (white arrow) demonstrates thickening suggesting a small hemorrhage, less pronounced than the left.

On POD 8, he presented with severe abdominal pain and CT demonstrated an ileus without evidence of postoperative complication. He was readmitted and gradually improved over the next 3 days but continued to experience intermittent abdominal pain and inconsistent bowel function. Therefore, on POD 11 a CT was repeated, demonstrating a new high density 2.6 × 2.6 cm left adrenal collection consistent with subacute hemorrhage (Fig. 1). The right adrenal gland appeared more prominent although not as striking as the left. Findings were consistent with BAH.

Although normotensive, he had significant electrolyte abnormalities including hyponatremia (128 mEq/L) and hypochloremia (96 mEq/L). Serum potassium and magnesium were normal. A corticotropin stimulation test (CST) revealed no elevation in cortisol after 30 and 60 min with an elevated adrenocorticotropic hormone (ACTH) twice the upper limit of normal, consistent with AI.

He received hydrocortisone 25 mg IV and the following day he was transitioned to a hydrocortisone 20 mg per oral (PO) morning dose and 10 mg PO evening dose. He remained normotensive and bowel function normalized. At his 2-week post-operative visit, his abdominal pain had resolved. Repeat CST after 6 weeks again failed to stimulate cortisol. He was continued on hydrocortisone at a reduced dose and care was established with an endocrinologist.

## DISCUSSION

Postmortem studies have reported the incidence of AH to range from 0.14 to 1.8%, with higher prevalence in critically ill patients [1–3]. Etiologies implicated in AH include sepsis, trauma, surgery, coagulopathy and neoplasm [1, 2]. Risk increases with underlying adrenal pathology as well as age above 55 and intensive care unit stays over 14 days [9]. BAH can lead to life-threatening AI, in which ˃90% of the adrenal cortex is nonfunctional. However, AI in the setting of AH remains uncommon, with reported prevalence <0.01% [10].

The pathophysiology of AH involves a prothrombotic state; adrenal vein thrombosis causes increased intra-gland pressure. Trauma and surgery may also cause vasoconstriction due to catecholamine release [2]. Cytokines such as tumor necrosis factor α, interleukins β, 6 and 12, have all been implicated in stress response leading to coagulation [10, 11]. The singular venous outflow tract coupled with significant vascularization subsequently increases risk of hemorrhage.

Presentation of BAH is often nonspecific, making diagnosis difficult [8]. Presenting symptoms and findings include abdominal/flank pain, fever, leukocytosis or anemia secondary to hemorrhage [10]. Serum cortisol levels, corticotropin, ACTH stimulation testing and electrolytes (sodium and potassium to assess mineralocorticoid function) may or may not be abnormal [12]. Imaging facilitates diagnosis of AH. Color Doppler ultrasound may demonstrate decreased adrenal blood flow, though identification is often challenging [1]. CT is the ideal imaging choice, and radiographic findings may include train tracking or low attenuation indicating decreased gland perfusion and expansion of the glands in cases of prolonged bleeding [1, 13]. Magnetic resonance imaging, though not routine, may be used when CT is not acceptable (e.g. pregnancy) or when more detail is desired [1].

Immediate intervention should be initiated, including corticosteroid administration, fluid resuscitation and correction of electrolyte imbalances [2]. Hypotension may require intravenous fluids or ionotropic support. In severe cases, endovascular embolization or laparotomy may be necessary when hemodynamic stability cannot be achieved by non-operative measures [1].

BAH has previously been described following abdominal surgery [4, 5, 8, 12]. In published reports of BAH after cholecystectomy, and after partial colectomy for adenocarcinoma, primary antiphospholipid antibody syndrome has been implicated [4, 5]. Additionally, BAH has been described after liver abscess drainage and perforated diverticulitis [6, 7]. This is the first report of BAH post- Whipple procedure.

In the case of our patient, aside from abdominal surgery and age over 55, he had no risk factors for AH such as adrenal masses or hypercoagulability. Similarly to prior reports, he presented with abdominal pain, electrolyte abnormalities and was found to have BAH on CT; AI was subsequently confirmed with CST and treated with steroids.

## CONCLUSION

Postoperative BAH is a rare but serious occurrence with unclear mechanism. We present the first incident of BAH after pancreaticoduodenectomy at our institution and in published literature, which was diagnosed and treated expeditiously resulting in a good clinical outcome. This case highlights the need to suspect BAH in the setting of vague postoperative symptoms to facilitate hasty diagnosis and treatment given the potential mortality.

## CONFLICT OF INTEREST STATEMENT

The authors have no conflicts of interest to disclose.

## FUNDING

None.

## DATA AVAILABILITY

No publicly available datasets were used in the writing of this manuscript.

## ETHICAL APPROVAL

Signed informed consent was obtained from the patient for the use of data and images in this manuscript. A copy may be provided upon request.
